# Roles of Reactive Oxygen Species in Cardiac Differentiation, Reprogramming, and Regenerative Therapies

**DOI:** 10.1155/2020/2102841

**Published:** 2020-08-28

**Authors:** Jialiang Liang, Min Wu, Chen Chen, Mingjie Mai, Jinsong Huang, Ping Zhu

**Affiliations:** Guangdong Cardiovascular Institute, Guangdong Provincial People's Hospital, Guangdong Academy of Medical Sciences, Guangzhou, Guangdong 510100, China

## Abstract

Reactive oxygen species (ROS) have been implicated in mechanisms of heart development and regenerative therapies such as the use of pluripotent stem cells. The roles of ROS mediating cell fate are dependent on the intensity of stimuli, cellular context, and metabolic status. ROS mainly act through several targets (such as kinases and transcription factors) and have diverse roles in different stages of cardiac differentiation, proliferation, and maturation. Therefore, further detailed investigation and characterization of redox signaling will help the understanding of the molecular mechanisms of ROS during different cellular processes and enable the design of targeted strategies to foster cardiac regeneration and functional recovery. In this review, we focus on the roles of ROS in cardiac differentiation as well as transdifferentiation (direct reprogramming). The potential mechanisms are discussed in regard to ROS generation pathways and regulation of downstream targets. Further methodological optimization is required for translational research in order to robustly enhance the generation efficiency of cardiac myocytes through metabolic modulations. Additionally, we highlight the deleterious effect of the host's ROS on graft (donor) cells in a paracrine manner during stem cell-based implantation. This knowledge is important for the development of antioxidant strategies to enhance cell survival and engraftment of tissue engineering-based technologies. Thus, proper timing and level of ROS generation after a myocardial injury need to be tailored to ensure the maximal efficacy of regenerative therapies and avoid undesired damage.

## 1. Introduction

Myocardial infarction (MI) is an anemic infarct disease associated with cell death of myocardium and frequently causes heart failure or cardiac arrest [1]. Recently, the promising therapeutic strategies have emerged for regeneration of cardiomyocytes (CMs) or remuscularization of the myocardium in MI [2], including induction of endogenous CM proliferation, direct reprogramming of nonmyocytes to CMs, and transplantation of pluripotent stem cell- (PSC-) derived CMs. Although these studies have demonstrated substantial potentials of *in vitro* and *in vivo* CM regeneration, several notable challenges remain to be addressed before translation to a clinical setting. For instance, insufficient long-term engraftment and integration with host tissue after transplantation remains a critical hurdle for using PSC-CMs in regenerative therapy [3]. Other issues including low regeneration efficiency, immaturity, and tumorigenic risk would compromise the therapeutic effects of new regenerative approaches [2, 4]. Therefore, it is important to converge various biochemical strategies with methods developed for regeneration of functional CM to overcome these challenges [5].

Current protocols of CM regeneration have been developed based on activating the embryonic cardiomyogenesis-induced signaling pathways and gene regulatory networks [6]. Most studies of CM regeneration are focusing on the contributions of transcriptional mechanisms including gene programming, epigenetic chromatin modifications, and biochemical differentiation cues [7]. Energy metabolism is central to mammalian heart development and function, and metabolic processes can be modulated to support the contractile apparatus of regenerated CMs [8]. The change in energy metabolism impacts the ability of stem cell self-renewal, differentiation, and cell fate decision [9]. Although the coordination of genetic networks with developmental bioenergetics is critical to CM phenotype specification, the underlying metabolic mechanisms that drive cardiac differentiation are not fully known.

The metabolic processes in heart development and disease are regulated by redox signaling through the direct effects of O_2_ levels and the byproduct-reactive oxygen species (ROS) [10]. Emerging evidence shows that the production and signaling of ROS plays an important role in heart development and pathogenesis of cardiovascular disease [11, 12]. ROS serve as an important driver of cell cycle arrest in postnatal CMs, and the mechanisms of CM proliferation have been summarized comprehensively [13, 14]. In this review, we discuss the current state of the art in effect of redox signaling on the strategies of myocardial regeneration including PSC-CM differentiation and cardiac reprogramming. In addition, we focus on the effect of ROS on PSC-CM engraftment in the host environment and highlight the importance of antioxidant approaches for enhancing efficacy of cell therapy.

## 2. Generation and Function of ROS

Here, we briefly outline the sources, forms, and functions of ROS related to cardiac biology.

### 2.1. Main Sources of Cellular ROS

Oxidation and reduction (redox signaling) induce changes in structural and functional characteristics of molecules or proteins by loss or gain of an electron, thus mediating transmission and amplification of metabolic signals. The major molecules that participate in redox signaling are ROS that are byproducts of the metabolism of oxygen such as superoxide, hydrogen peroxide, and hydroxyl radical [15]. Cellular ROS mostly originate from superoxide O_2_^·-^ produced by nicotinamide adenine dinucleotide phosphate (NADPH) oxidases (NOXs), the electron transport chain (ETC) in the mitochondria, or the nitric oxide synthases (NOSs).

The NOX family includes seven NOX isoforms with distinct catalytic subunits and they are crucial regulators of redox signaling in multiple body systems and organisms [16]. NOX enzymes can transfer electrons from NADPH to oxygen across biological membranes to produce ROS in both phagocytic and nonphagocytic cells [16, 17]. Mitochondrial ETC transfers electrons from NADH (nicotinamide adenine dinucleotide hydrogen) and succinate along a controlled redox path during respiratory ATP synthesis. However, the electron flow in ETC is an imperfect process, and occasionally oxygen molecules may undergo one- or two-electron reduction reactions to form ROS [18]. Depending on mitochondrial states of respiration, complexes I and III of the ETC may contribute to ROS production through leakage of electrons [18, 19]. NOSs catalyze the conversion of L-arginine to L-citrulline and NO, but can become uncoupled under pathological conditions and switch to ROS production [20].

### 2.2. ROS Exert Different Physiological and Pathological Functions

ROS can be classified depending on their chemical properties into two groups: one-electron oxidants (e.g., free radical O_2_^·-^ and HO^·^) and two-electron oxidants (e.g., nonradical H_2_O_2_) [21]. Superoxide O_2_^·-^ can diffuse within a cell with a relatively longer half-life as compared with other radicals but is neither a strong oxidant nor a powerful reductant [22]. Hydrogen peroxide H_2_O_2_ is stable, diffuses within and between cells, and can function as a signaling molecule or second messenger in the regulation of a variety of biological processes [23]. Hydroxyl radical HO^·^ is formed from H_2_O_2_*via* Fenton chemistry in the presence of Fe^2+^. HO^·^, the most reactive ROS, is responsible for DNA damage, oxidative stress, and lipid oxidation, but its short half-life (10^−9^ s) restricts its damaging effects [24, 25]. Therefore, H_2_O_2_ appears to be a critical ROS molecule in redox-dependent signal transduction.

It is known that a physiological H_2_O_2_ flux activates signaling pathways by reversible oxidation of effector proteins. H_2_O_2_ oxidizes the thiol side chain of cysteine residues of the targeted functional motifs [26]. The cysteine residues are modified with highly susceptible thiolate anions under physiological condition, while oxidation of these anions into sulfenic forms can change the activity and function of proteins such as protein tyrosine kinases and transcription factors (TFs) [27, 28], thereby modulating the downstream gene expression and cell behaviors.

The borderline between “oxidative eustress” (beneficial responses) and “oxidative distress” (deleterious responses) in different pathophysiological settings is highly context dependent and remains to be clearly characterized in health and disease [29]. When ROS concentrations remain at physiological levels, they are indispensable in maintaining cell signaling and redox homeostasis. However, excessive production of ROS or oxidative stress has been associated with disease pathogenesis including cardiovascular disease and cancer [30]. ROS regulate diverse processes such as cell death, calcium handling, and cardiac hypertrophy involved in the pathophysiology of heart failure [31].

ROS levels are influenced not only by their generation rate but also by ROS-scavenging systems or antioxidants. Endogenous antioxidant defense system exists to detoxify ROS, repair oxidative damage, and maintain redox homeostasis [32]. Specific endogenous antioxidants such as catalase, peroxiredoxins, thioredoxin, and glutathione peroxidases can prevent potential damage of overoxidation by H_2_O_2_ [33, 34]. Our previous study also demonstrated that H_2_O_2_-induced CM hypertrophy was improved by activation of antioxidant heme oxygenase-1 (HO-1) [35].

In addition, the compartmentalization and temporal profiles of ROS need to be considered to interpret the consequences of downstream signaling cascades. For instance, elevated mitochondrial ROS is a principal source of oxidative stress leading to arrhythmias and contractile dysfunction in heart failure, and reduction of mitochondrial ROS (rather than cytoplasmic ROS) can prevent and reverse electrical instability and sudden cardiac death [36]. Thus, the physiological roles of ROS and their toxic effects are complicated, which are influenced by a multitude of factors including concentration, source, distribution, and type of ROS. We discuss the complex roles of ROS, H_2_O_2_ in particular, in CM differentiation and heart regenerative therapy below.

## 3. ROS Mediate Cardiac Differentiation of PSCs

PSCs including iPSCs (induced pluripotent stem cells) and ESCs (embryonic stem cells) have emerged as one of the promising cell resources used to differentiate into functional CMs for heart regeneration [37]. Activation of embryonic signaling pathways including Activin, TGF-*β*, Wnt, and BMP is essential for development of CM lineage [38]. Multiple complex interactions between these conserved signaling pathways control the initial differentiation, proliferation, and maturation of myocardium to establish the cardiovascular system [38]. The delineation of specific redox-sensitive pathways and mechanisms that contribute to different components of CM regeneration processes may facilitate to fine-tune existing protocols or devise novel strategies in heart disease modeling and therapy.

New CMs can be generated from mesodermal progenitors during spontaneous differentiation (embryoid body (EB) formation or a monolayer induction) of PSCs by using growth factors and small molecules mimicking developmental signals [39, 40]. For stem cell culture and maintenance, ROS scavengers or antioxidant supplements are extensively used to prevent cellular oxidative stress [41]. However, *β*-mercaptoethanol and other thiol-based antioxidant supplements may cause changes to cellular redox state and then reduce the cardiogenic potential of stem cells [42]. The molecular mechanisms involved in metabolism and ROS regulation of PSC differentiation are still poorly understood and merit further investigation to optimize stem cell culture methods.

### 3.1. Generation of ROS in Early Differentiation Stage

**Figure 1:**
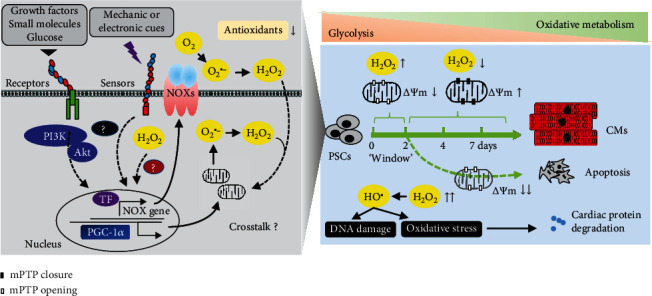
ROS are important for initial stage of differentiation but dispensable for the late stage. ROS are generated by multiple pathways and involved in differentiation of PSCs in response to developmental cues. After closure of mPTP, ROS are decreased and redox signaling is set for further differentiation and functional maturation, while excessive ROS levels would inhibit this process through increased oxidative stress and degradation of structural proteins, eventually leading to apoptotic cell death. NOXs: NADPH oxidases; TF: transcription factor; PGC-1*α*: peroxisome proliferator-activated receptor *γ* coactivator 1*α*; mPTP: mitochondrial permeability transition pore; PSCs: pluripotent stem cells; CMs: cardiomyocytes.

Accumulating evidence shows that intracellular ROS are a critical signal to trigger CM differentiation of stem cells (Figure 1). The intracellular ROS level was increasing in early stage of mouse ESC differentiation [43]. The differentiation cues (e.g., growth factors, small molecules, mechanical stimulus, and electrical fields) were found to increase ROS level in ESCs, while cardiac lineage formation would be impaired by inhibition of ROS-generating pathways or ROS activity [44–46].

Compared to differentiated cells, PSCs have few immature mitochondria (that are globular in shape with poor cristae structure) and mostly rely on glycolysis to meet their energy demands [47, 48]. Therefore, cardiac specification and excitation-contraction coupling require a switch of glycolytic metabolism towards more efficient mitochondrial oxidative metabolism in PSCs. The energetic switch during differentiation of ESCs was programmed by rearrangement of the metabolic transcriptome (encoding enzymes of glycolysis, fatty acid oxidation, the Krebs cycle, and the ETC) and development of a mature mitochondrial network [49]. ROS are subsequently generated during oxidative metabolism in redox regulation of mitochondrial biogenesis and promote cardiac differentiation and maturation [50]. Thus, ROS generation is potential crosstalk between genetic and metabolic signaling in directing cell fate.

The mechanisms underlying ROS generation remain poorly known in current studies of initiating cardiac differentiation of PSCs. A cytokine-PI3-kinase-NOXs cascade was reported as an initial signal of ROS upregulation in cardiac differentiation of mouse ESCs [43, 45], suggesting the role of ROS as intracellular second messengers. Additionally, stimulation of fatty acid metabolism by activation of peroxisome proliferator-activated receptor-*α* may be an upstream signal of NOX4-induced ROS generation in mouse ESCs, while mitochondrial electron transport was not involved in this process [51]. Mechanical strain-NOXs, metabotropic glutamate receptor 5, and the PI3K/AKT pathway may also contribute to ROS generation in cardiomyogenesis of ESCs [52–54]. Other studies showed a high expression level of NOX4 in mouse ESCs and demonstrated it as an important source of ROS signals involved in cardiomyogenesis by using siRNA approach [55]. NOX4-induced ROS was also an important signal of differentiating cardiac progenitors under stimulation of magnetic fields [56].

While most of the above studies involve activation of NOX4, ROS derived from mitochondria also play an important signaling role in differentiation and maturation. Specific antagonists had been used to demonstrate an essential role of complex III activity of the mitochondrial ETC in cardiac differentiation and calcium oscillations [57]. In mitochondria of cardiac myocytes, complex III is the principal site for ROS production during the oxidation of complex I substrates [58]. Importantly, a high glucose concentration had been shown to promote cardiac differentiation of ESCs *via* mitochondrial ROS generation [59]. Temporally reduced antioxidant activity of peroxiredoxin-2 *via* nitrosylation can cause transient endogenous ROS accumulation and promote ESC-derived cardiomyogenesis [60]. During cardiac differentiation of human ESCs, PGC-1*α*-dependent mitochondrial biogenesis was associated with increased ROS levels in the CM population [61]. Therefore, cellular ROS are tightly regulated by a variety of proteins involved in the redox regulation of PSCs undergoing a metabolic switch when they differentiate.

ROS may be differently generated in multiple subcellular compartments in targeted cells. Communications between these distinct sites of ROS generation are also functionally relevant to cardiac differentiation. NOX4 can be activated by mitochondrial ROS in differentiated ESCs under the high glucose condition, suggesting an integrated signal between NOXs and mitochondrial ETC [59]. Moreover, a feed-forward regulation of ROS generation was shown by H_2_O_2_-induced NOX4 gene expression in cardiac differentiation [62]. Intriguingly, an increasing level of ROS can lead to further release of mitochondrial ROS, termed ROS-induced ROS release, which propagates and amplifies ROS production and effects in cardiac myocytes [63], although this remains undetermined in cardiac differentiation.

The location of ROS generation should be considered when interpreting their effects. Although instructive, the antioxidant compounds do not readily identify the source of ROS due to low specificity. The dynamics of H_2_O_2_ metabolism can be assessed by the use of fluorescent probes and other redox-sensitive tools [64]. H_2_O_2_ release and cell distribution can be visualized by new ratiometric reporters that have been targeted to subcellular compartments [65]. These molecular tools will be a more specific system for *in vivo* monitoring of cardiac redox signaling and heterogeneity of individual cell responses to oxidants.

### 3.2. Continuous Exposure to ROS Inhibits Cardiomyogenesis

The physiological range of H_2_O_2_ concentrations was estimated to be between 1 and 10 nM, but it depends on several parameters including cell type and developmental stage [66]. Exogenous H_2_O_2_ is a useful tool to determine the direct contribution of ROS in CM differentiation. Stimulation of cardiomyogenesis by exogenous H_2_O_2_ (10 nM) was showed to increase the number of beating EB containing CMs and the expression of cardiac genes at 2-3 induction days [43, 55, 62]. Several cardiogenic TFs and cytokines were upregulated by addition of H_2_O_2_ in ESCs [67].

In addition to ROS sources, the role of ROS in cardiac differentiation is dependent on metabolism phases and redox balance. Continuous exposure to ROS at a high concentration may overwhelm the antioxidative capacity of cells, thereby exerting a detrimental effect on cell differentiation. Indeed, exogenous H_2_O_2_ (100 nM) was showed to inhibit the beating activity of EBs from day 5 to 12 [68]. Excessive H_2_O_2_ levels (1 *μ*M) can reduce and degrade Gata4 protein in P19 stem cells [69]. Moreover, increase of intracellular ROS level was responsible for inhibitory effect of valproic acid on cardiomyogenesis [70]. The enforced expression of the pyruvate dehydrogenase phosphatase catalytic subunit 1 gene increased mitochondrial ROS levels in ESCs and inhibited cardiac differentiation [71].

These data suggest that a particular window of “cardiopoietic programming” [72] may exist where a proper level of ROS is important for cardiac differentiation during early stages. During the early period of cardiac differentiation, a high ROS level and low ATP production from immature mitochondria of PSCs may help themselves (or regenerative cells) to adapt to the stress of metabolic switch. After metabolic demand is fulfilled, activation of endogenous antioxidant defense will decrease ROS level to avoid excessive oxidative stress on genetic programming of further CM differentiation and maturation (Figure 1).

Accumulating evidence points out that the redox signaling is associated with mitochondrial permeability transition (MPT) regulating myocyte differentiation and maturation. MPT is caused by the opening of mitochondrial permeability transition pores (mPTP) in the inner mitochondrial membrane. mPTP opening can couple to mitochondrial ETC-dependent ROS production in unstressed cells [73], while mechanisms by which mPTP regulates ROS remain to be determined. Importantly, a study of heart development showed that mPTP opening was nonpathologic in embryonic cardiac myocytes (E9.5) with immature mitochondrial structure and function, low ATP production, and high ROS levels [74]. Differentiation of embryonic CMs was accelerated after closure of mPTP companied with decreased ROS levels, whereas concurrent treatment with oxidant and mPTP blocker inhibited differentiation [74]. Therefore, the beneficent effect of ROS in the window of “cardiopoietic programming” would be offset after closure of mPTP.

Recently, some mPTP inhibitors have been assessed for inducing cardiac differentiation. mPTP inhibition by cyclosporine-A increased ROS generation, but addition of antioxidants rather than prooxidant can enhance cardiomyogenesis [75]. Prolonged closure of mPTP with cyclosporine-A in human iPSC-derived endothelial cells resulted in more mature mitochondria, prevention of ROS leakage, and functional improvements [76]. These studies suggested that the redox signaling is a cardiogenic regulatory factor lying the downstream of mPTP inhibition. The approaches relying on manipulation of redox status should be dependent on monitoring the mode of mPTP.

There are several common features (e.g., cytochrome c release and caspase activation) that govern cell differentiation and apoptosis [77, 78]. MPT and ROS are known to involve in the etiology of several pathological conditions related to necrosis and apoptosis [79], while they can trigger cell differentiation as discussed above. Basic ROS activity contributes to cell differentiation but can induce caspase-dependent apoptosis once the oxidative stress exceeds a certain threshold [80]. Lower levels of ROS, loss of one p53 isoform, and reversible loss of the mitochondrial membrane potential were observed in the differentiating cells as compared to the apoptotic cells that were induced by doxorubicin treatment (an antitumor agent or useful tool with cardiotoxicity), although these features were absent in undifferentiated ESCs [81]. This study indicated that the timing, intensity, and reversibility of activation of mitochondrion-dependent apoptotic pathway may determine whether a cell dies or differentiates.

### 3.3. ROS Regulate Cardiac Gene Transcription and Expression

**Figure 2:**
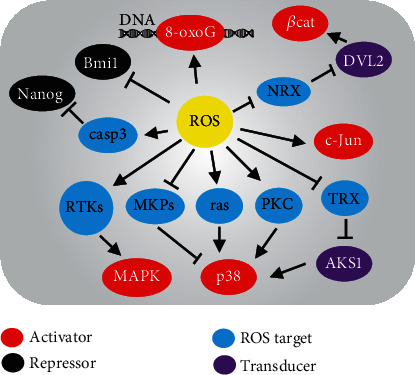
A possible network of molecular events targeted by ROS related to cardiac differentiation pathways. The activators or repressors of cardiac gene expression can be directly or indirectly regulated through ROS modifying the redox-sensitive molecules. 8-oxoG: 8-oxoguanine; *β*cat: *β*-catenin; casp3: caspase3; DVL2: dishevelled segment polarity protein 2; NRX: nucleoredoxin; TRX: thioredoxin; ASK1: apoptosis signal-regulating kinase 1; PKC: protein kinase C; RTKs: receptor tyrosine kinases; MAPKs: mitogen-activated protein kinases; MKPs: MAPK phosphatases.

ROS have been considered as critical small-molecule messengers in cell signaling transduction. Several signal transducers are redox-sensitive and can be reversibly or irreversibly modified by ROS, providing a link with the control of gene expression [82]. Principal modifications are selective oxidation or nitrosylation of key redox-sensitive cysteine residues in kinases with low ionization pKa (4-5 *vs.* 8.5 in nonreactive cysteines of most other proteins) [83]. Cysteine oxidation results in either inhibition or activation of targeted molecules depending on the tertiary structure [83]. Furthermore, ROS have been implicated in modulating epigenetic pathways including histone modifications, DNA modifications, expression of noncoding RNAs, and ATP-dependent chromatin remodeling in cardiovascular diseases [84]. Herein, we discuss the direct targets of ROS involved in the mechanisms of cardiac differentiation and heart regeneration (Figure 2).

In response to differentiation cues such as growth factors, the downstream cell signaling pathways will be activated before the gene transcription determining cardiac lineage [85]. Tightly controlling phosphorylation of mitogen-activated protein kinase (MAPK) is important for early mesoderm and subsequent CM formation [86]. ROS were shown to enhance differentiation of human ESCs into bipotent mesendoderm *via* the activation of MAPK family [87]. The phosphorylation of p38 MAPK was inhibited by knockdown of NOX4 and nuclear translocation of Mef2c was prevented, thereby reducing cardiac differentiation [55]. Activation of p38 MAPK was eliminated by an antioxidant in ESCs, and p38 phosphorylation may provide a checkpoint during mesodermal differentiation to the cardiac lineage [59]. These studies suggested that activation of p38 MAPK was closely related to high ROS levels.

In contrast, activation of p38 MAPK mediated by ROS was involved in inhibiting cardiac differentiation of murine ESCs [88], suggesting that the effects of p38 MAPK may be different in distinct timing of differentiation. It remains unknown how ROS can interact with p38 MAPK signaling during cardiogenesis. Oxidative modifications of upstream signaling proteins or receptor kinases by ROS may be a plausible mechanism for activation of the MAPK pathways [89]. Apoptosis signal-regulating kinase 1 (ASK1) was an upstream protein of p38 MAPK and bound to reduced thioredoxin in unstressed HEK293A cells, while thioredoxin can be oxidized upon oxidative stress and disassociate from ASK1, thereby leading to p38 phosphorylation *via* oligomerization of ASK1 [90]. Alternatively, degradation or inactivation of MAPK phosphatase by ROS-related ubiquitin-proteasome system may contribute to activation of the MAPK pathways in ESCs and other cells [91, 92]. Therefore, MAPKs might not be directly redox-sensitive but instead rely on ROS-mediated upstream proteins such as ras and PKC [93]. These potential mechanisms of ROS-related pathways need to be further determined in the setting of cardiac differentiation.

Cardiac commitment of PSCs is controlled by the regulatory network of TFs such as Nkx2.5, Gata4, and Tbx5 [6, 85]. Although these TFs might not be directly targeted by ROS, their transcription can be regulated by other epigenetic modulators or constitutively active TFs (e.g., AP1 and HIF1*α*) that are ROS-sensing in vascular cells [94]. Expression of earliest cardiogenic TFs such as Gata4 and Mef2c was dependent upon Nox4-generated ROS that activate redox-sensitive TFs including c-Jun in P19 stem cells [95]. Moreover, extrinsic ROS can enhance the redox-sensitive caspase-mediated degradation of Oct4 and Nanog (pluripotent factors), thereby activating Gata4 and Nkx2.5 promoters that were repressed by Nanog/Hdac4 complex in P19 stem cells [69]. Interestingly, an increase of ROS due to removal of antioxidant in medium can induce epigenetic DNA modifications (such as 8-oxoG) on Tbx5 promoter, leading to Tbx5 activation that enhanced cardiac differentiation of ESCs [96]. Bmi1 is an epigenetic repressor silencing cardiac genes in steady state of cardiac progenitors, while ROS and oxidative damage induced Bmi1 delocalization from canonical DNA targets, therefore triggering an imbalance toward upregulation of differentiation-related genes and downregulation of stemness-related genes in cardiac progenitors [97]. In neural progenitor cell lines, ROS may induce dissociation of redox-sensitive targets such as nucleoredoxin from dishevelled complex that was responsible for activation of the Wnt/*β*-catenin cascade in transcription of differentiation-related genes [98]. Although different cell models including ESCs have been tested, ROS may regulate downstream gene expression through a common mechanism targeting the transcription-related factors.

The above mechanism studies suggest that identification of redox-sensitive targets helps to delineate how ROS or oxidative stress contributes to cell fate decision. New methods are therefore needed to screen ROS targets and verify their redox functions in various models of cardiomyogenesis and heart regeneration on a global scale. For instance, cysteine reactivity in response to oxidative modifications can be labeled using chemical probes and further assessed by quantitative mass spectrometry in targeted proteins or in a whole proteome scale [99, 100]. In addition to protein assays, several methods have been developed using next-generation sequencing to assess the genome wide distribution of oxidative DNA modifications [101]. Importantly, several computational tools and databases have been developed for analysis of redox-sensitive cysteines and annotation of ROS-related proteins and peroxidase families [102, 103]. Thus, these chemical-genetic methods enable detailed characterization of protein or DNA modifications that are targeted by ROS in the redox environment related to CM regeneration.

## 4. Unexploited Role of ROS in Direct Cardiac Reprogramming

Transdifferentiation is a new paradigm that has been devised to generate cardiac lineage-specific cells directly from somatic cells, by combining transient overexpression of the cardiac specific TFs. The retroviral transfections of Gata4, Mef2c, and Tbx5 (or with Hand2) reprogramed mouse postnatal cardiac or skin fibroblasts directly into CM-like cells (termed induced CMs (iCMs)), but with low efficiency [104–106]. The TF overexpression was an inefficient method to induce cardiac reprogramming, and the infected cells lacked some molecular and electrophysiological phenotypes of mature CMs [107]. Therefore, researchers are exhibiting tremendous enthusiasm and interest in the quest to elucidate the mechanisms of iCM generation and further enhance reprogramming efficiency. The current progress in this field has been summarized in other reviews [108, 109].

Yet, it remains unknown whether ROS are involved in the process of direct cardiac reprogramming. A preliminary study showed that the treatment of vitamin E nicotinate (an antioxidant) facilitated application of direct cardiac reprogramming approach to repair heart damage *in vivo* [110]. Further investigation should determine whether the observed effects were related to the elimination of ROS or redox imbalance in iCMs or injured host CMs. Exogenous ROS incubation, use of redox-sensitive probes, treatment of antioxidants in different induction timing, and loss-of-function studies of ROS-associated genes would be helpful strategies to address the unexplored role of ROS in both *in vitro* and *in vivo* direct cardiac reprogramming.

The studies of ROS in induced pluripotency reprogramming may bring new insights into genetic resetting during direct cardiac lineage conversion. NOX expression and ROS generation were increased in the early stage of iPSC reprogramming, whereas antagonism of ROS using antioxidants or knockdown of NOXs decreased reprogramming efficiency [111]. Excessive ROS generation impaired iPSC generation, and antioxidant enzymes such as Gpx2 and Nrf2 were upregulated in the late phase of reprogramming [111, 112]. Therefore, these data indicate that the kinetics and intensity of redox signaling is critical for efficient cell reprogramming. Importantly, short-term opening of mPTP has been found during the early stage of somatic cell reprogramming into iPSCs, as companied with activation of mitochondrial ROS [113]. Furthermore, ROS generation triggered by activation of innate immune signaling is required for pluripotent reprogramming and lineage transdifferentiation [114].

The precise mechanisms of direct cardiac reprogramming are not well understood. Recently, next-generation sequencing techniques have been employed not only to decipher the transcriptional mechanisms of cardiac TFs but also to uncover the dynamic process of cell fate reprogramming in a genome-wide scale or at a single-cell level [115–117]. These data suggest that innate immune signaling is critical for cardiac fate acquisition at early stage and cell cycle exit is essential for successful reprogramming. It is conceivable that immune response genes can be activated due to the common use of viral vectors for reprogramming gene delivery [118, 119]. For instance, expression of Toll-like receptor 3 (an immune regulatory gene) contributed to human cardiac reprogramming through impacting DNA methylation status of cardiac loci [115]. Given that ROS can interact with innate immune receptors including Toll-like receptors and NOD-like receptors [120], it is likely that ROS are an important signal during the early stage of cardiac reprogramming and redox balance ensures the further functional maturation of iCMs, which is similar to cardiac differentiation as discussed above. However, it is unknown whether the innate immune pathways are still reactivated in alternative, nonviral reprogramming approaches such as chemically induced CM-like cells [121]. Despite the complexity, the ultimate goal of cardiac TFs or reprogramming factors is to convert the fibroblasts to contracting muscle cells with a high metabolic demand. Based on gene expression of metabolic enzymes, iCMs utilized fatty acid oxidation as the main pathway, which was distinguishable from iPSC-CMs primarily using glycolysis [122]. All above findings encourage further investigation of ROS in the mechanisms of cardiac reprogramming with respect to chromatin accessibility changes, innate immune response, cell cycle regulation, and metabolic switch.

## 5. ROS Affect Regenerative Therapy in the Infarcted Heart

**Figure 3:**
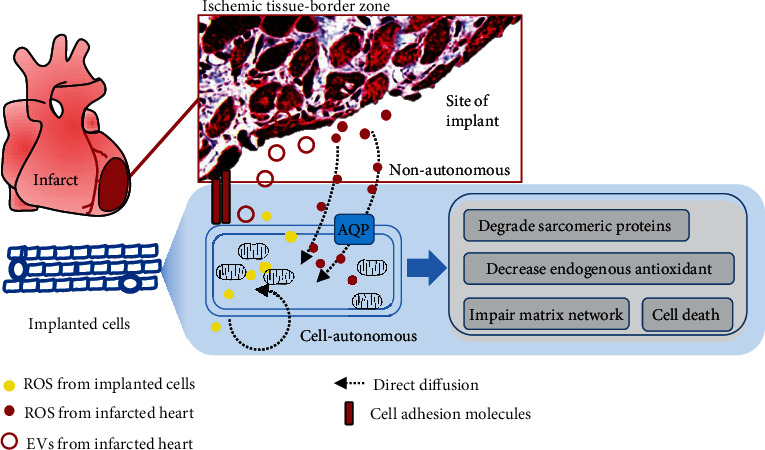
Potential interactions between the ischemic heart and implanted cells contribute to low engraftment efficiency. When stem cells or PSC-CMs are implanted, intracellular ROS would be increased and induce cell death in a cell-autonomous manner in response to the hypoxic microenvironment. Paracrine effects of host's ROS are involved in regulation of the graft cell fate and may lead to engrafted cell death in a nonautonomous manner. EVs: extracellular vesicles; AQP: aquaporin.

Currently, PSCs are the main cell sources that can definitively generate cardiovascular cells (seed cells) in high quantities for MI therapy using cardiac tissue engineering [123]. However, insufficient integration of transplanted cells with ischemic tissue remains a major hurdle for clinical translation of using engineered heart tissues (EHTs) in regenerative therapy. Understanding of the healing process of MI, including inflammatory, proliferative, and maturation phases, is important for design and timing selection of cell transplantation in patients. There exists a potential feedback loop (cell-cell interaction) between the host infarcted myocardium and the engraftment of implanted cells, as discussed by us [124]. In this section, we integrate the current evidence to speculate how ROS affect the cell survival and functional engraftment of implanted or regenerated CMs in the infarcted heart (Figure 3).

### 5.1. A High Level of Intracellular or Extracellular ROS Harms Graft Cell Survival

Clinical application of stem cell therapies requires large-scale cell culture technologies such as bioreactors that allow for conditional manipulations of the survival, differentiation, and maturation of PSC-CMs [125]. Maintenance of low cellular H_2_O_2_ concentration may facilitate *in vitro* maturation of PSC-CMs [126]. However, PSC-CMs appear to be particularly sensitive to hypoxia and nutrient deprivation-induced cell death associated with increased ROS formation and modulation of key nutrient sensors [127]. A gradual cessation of contractility with increased intracellular ROS and loss of calcium transients was found in mouse PSC-CMs after short-term exposure to monochromatic light [128]. It is likely that ROS-induced protein glutathionylation contributes to a loss of myofibril integrity and degradation of sarcomeric proteins in CMs [129]. Cellular ROS are substantially elevated in cardiovascular cells during ischemia and reperfusion procedure and also involved in the post-MI remodeling of heart failure [36, 130]. Excessive ROS generation depletes endogenous antioxidant defenses in the ischemic heart and primes the cell for oxidative damage at reperfusion [130]. ROS also can persistently impair myocardial matrix network by nonenzymatic protein degradation and modification or activating specific proteolytic enzymes [130]. Therefore, maintenance of redox homeostasis through reduced intrinsic ROS generation and increased antioxidant defense mechanism may promote therapeutic efficacy of cardiac cell replacement approaches (see later).

Extracellular ROS and oxidative stress are critical components of harsh conditions in the infarcted myocardium. Despite the short half-lives, extracellular ROS likely participate in cell-cell communications at the site of ischemia. NOX isoforms are responsible for generation of superoxide (O_2_^·-^) toward intracellular or extracellular space and its autocrine or paracrine-like action [131]. Unlike superoxide free radicals (O_2_^·-^) with a negative charge, H_2_O_2_ is known as a membrane permeable molecule which can diffuse through the mitochondrial and cell membranes. Therefore, ROS can serve as a paracrine-diffusible signal to mediate nearby cells. For instance, H_2_O_2_ increased in the infarct core can diffuse into circulating cells and produce 3-nitrotyrosine that was cytotoxic and contributed to decreased recruitment of endogenous progenitor cells to the site of injury [132]. ROS generated in the infarcted heart can hinder the adhesiveness of injected cells *via* interference of focal adhesion molecules [133]. Interestingly, several aquaporins (water channels) have been identified to facilitate movement of H_2_O_2_ across cell membranes at a much higher rate than passive diffusion [134], but their roles remain largely unknown in cardiac cells or regenerated CMs.

### 5.2. New Insights of ROS in Intercellular Communications

New lines of evidence show that ROS signaling can be transferred in a diffusion-independent fashion from donor cells to nearby cells [135]. For instance, pericardial ROS were shown to directly modulate the expression of cell adhesion and cytoskeleton molecules that facilitate interaction between the pericardial cells and cardiac myocytes [136]. In addition, extracellular vesicles such as exosomes that derived from CMs in response to H_2_O_2_ were shown to exacerbate apoptosis of transplanted stem cells [137]. ROS were contained in microvesicles isolated from endothelial cells after hypoxia-reoxygenation, leading to apoptosis and oxidative stress in myoblasts [138]. A study of spinal injury brought a novel sight from finding of exosomal delivery of ROS-producing NOX2 to the injury site and triggering inflammation [139]. The potential mechanisms of ROS and their producers transferring through exosomes or microvesicles require further research in the setting of MI and cell therapies. Knowledge obtained from these studies helps to interpret a possibility that ischemic myocardium-derived ROS target engrafted stem cells or PSC-CMs in a paracrine manner *via* extracellular vesicles or free diffusion.

## 6. Convergences of Antioxidants and Cell-Based Therapy

**Figure 4:**
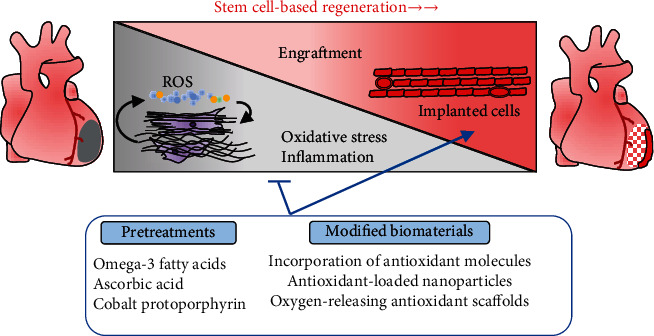
Overview of antioxidant approaches to enhance stem cell-based regeneration. Antioxidant strategies including pretreatments and modified biomaterials targeting the ROS signaling can be applied to enhance the engraftment of implanted stem cells or PSC-CMs.

When the oxidative insult overwhelms the endogenous antioxidant defense system during MI, a prolonged elevation in ROS levels leads to chronic inflammation with scarring and tissue dysfunction [140]. Therefore, proper timing and level of ROS generation after MI injury need to be tailored to ensure maximal efficacy in order to avoid undesired damage. Nevertheless, pharmacological interventions using nonselective antioxidants (e.g., vitamin C, vitamin E, and *β*-carotene) failed to show a significant impact on prevention or treatment of cardiovascular disease in trials [140]. Noneffective or harmful outcome of these antioxidants is likely owing to low drug specificity or disturbed the redox balance signaling. Moreover, systemic delivery of antioxidants might be limited by low bioavailability or low effective levels in the site of injury. To this end, stable materials are being developed for localized antioxidant activity. We could also take advantage of novel biomaterials using in cardiac tissue engineering to scavenge ROS, enhance graft survival, and achieve replenishment of the lost myocardium (Figure 4).

To obtain functional EHT for cell therapy, natural biomaterials or synthetic nanomaterials have been used to provide mechanical, electroactive support and generate 2D or 3D cardiac sheets [141, 142]. Nonetheless, oxidative stress would be generated due to a detrimental immune response to biomaterials at the site of implantation [143]. It remains challenging to identify biocompatible, biodegradable scaffolds that allow cell migration into infarct zone and protect cells against the oxidative stress. Antioxidants function in different mechanisms, such as free radical scavengers, singlet oxygen quenchers, inactivators of peroxides, chelators of redox metal ion, and quenchers of secondary oxidation products and inhibitors of prooxidative enzymes [144]. We focus on the antioxidative biomaterials that are cardiac-compatible and also show application potential to enhance graft cell survival in preclinical studies.

Recently, incorporation of small antioxidant molecules into polymeric scaffold is a straightforward means to retain the antioxidant activity. For instance, a degradable polyurethane backbone conjugating with ascorbic acid was shown to provide sustainable antioxidant properties and robust mechanical support for CM growth, which rescued CM death under oxidative stress [145]. Interestingly, incorporation of calcium peroxide into an antioxidant hydrophobic polymer can yield a 3D scaffold with a sustained oxygen release as well as attenuation of free radicals [146]. The development of scaffolds with oxygen-releasing and antioxidant properties will offer a unique solution to protect graft from hypoxia-induced cell death by providing sufficient oxygen and attenuating the oxidative stress during oxygen generation, leading to better survival of the critically perfused tissues [146]. Additionally, the antioxidant property of injectable hydrogel can be enhanced by structural introduction of antioxidants such as citric acid and glutathione, and their protective potential effects on graft cells have been determined in MI or oxidative stress models [147, 148]. Antioxidant-loaded nanoparticles can be embedded in hydrogel that possesses a highly porous structure, and this system might have an excellent biocompatibility to support the adhesion and survival of CMs for injectable cardiac tissue engineering [149, 150].

The development of scaffold-based cell delivery techniques is in the early stages for cardiac tissue engineering, and there are still opportunities to incorporate additional treatments to modulate the antioxidative and anti-inflammatory process. Pharmacological pretreatments (such as omega-3 fatty acids and cobalt protoporphyrin) have beneficent effects on survival of ESC-CMs as evidenced by upregulation of HO-1 and decreased ROS levels under oxidative or hypoxic conditions [151, 152]. Future study will reveal new targets and pharmacological compounds to enhance cell engraftment of EHTs after delineating the mechanisms by which the fate of transplanted cells is mediated by increased ROS or downregulated endogenous antioxidant system.

## 7. Perspectives and Conclusion

In light of the extensive impact of ROS on different aspects of cell differentiation and metabolic homeostasis, there has been continued interest in targeting ROS for therapeutic benefit in the development of heart regenerative medicine. The potential of redox signaling to promote or inhibit CM differentiation may depend upon the ROS source, cell context, and probably the magnitude of ROS generation. It should be noted that the beneficial or detrimental roles of ROS in this scenario do not necessarily need to be mutually exclusive. Cellular ROS may act through several targets and have diverse roles in different stages of cardiac differentiation, proliferation, and maturation. Stem cells are thought to maintain a low basal level of ROS for preserving their functions in quiescence, while increased ROS after differentiation can be countered by the antioxidant defense system to avoid sustained oxidative stress. Although mouse or human ESCs provide a unique experimental model to study the role of ROS and ROS-generating enzymes in the regulation of CM differentiation *in vitro*, it remains further investigation in human iPSCs to refine the methodologies regulating cellular redox states *via* metabolic modulations for translational research. Improvement in omic technologies, including genetic screening, single-cell approach, and large-scale profiling of redox-sensitive targets, will undoubtedly advance the understanding of the complexities of ROS and antioxidant pathways during cardiac differentiation and heart development. Additionally, the detailed role of ROS has not been determined in direct cardiac transdifferentiation (reprogramming). Further investigation of epigenetic mechanisms, innate immune response, and mitochondrial regulation will bring new insights into the field of metabolic reprogramming in order to enhance the CM conversion efficiency.

ROS also play a role in applications of cardiac regenerative therapies for MI treatment. Intracellular ROS are increased and induce cell death of implanted stem cells or PSC-CMs in a cell-autonomous manner in the ischemic microenvironment. Paracrine effects of host's cells on the site of implant also likely cause graft cell death in a nonautonomous manner due to uptake of transferred ROS. Yet, this remains to be elucidated to what extent potential paracrine mechanisms contribute to the low engraftment and survival rate of stem cell-based therapies. It will help to address this question by the gain- and loss-of-function studies of the relevant genes in ROS generation pathways in host and donor cells, respectively. This knowledge is important for the design and selection of antioxidant strategies for development of tissue engineering-based technologies. Natural or synthetic biomaterials with antioxidant activity have been used in tissue engineering scaffolds. Further optimization of cardiac tissue engineering needs in-depth evaluation of new biomaterials in regard to donor-host cell coupling, immunogenicity, antioxidant and anti-inflammatory activity, and mechanical and electronic properties. These antioxidant intervention approaches should ensure protecting against infarct expansion, ventricular rupture, and other potentially devastating post-MI complications and avoid disruption of other important signaling of self-healing processes when combining with stem cell-based technology.
